# Short-Term High Fat Intake Does Not Significantly Alter Markers of Renal Function or Inflammation in Young Male Sprague-Dawley Rats

**DOI:** 10.1155/2015/157520

**Published:** 2015-06-21

**Authors:** Catherine Crinigan, Matthew Calhoun, Karen L. Sweazea

**Affiliations:** School of Nutrition and Health Promotion, School of Life Sciences, Arizona State University, Tempe, AZ 85287, USA

## Abstract

Chronic high fat feeding is correlated with diabetes and kidney disease. However, the impact of short-term high fat diets (HFD) is not well-understood. Six weeks of HFD result in indices of metabolic syndrome (increased adiposity, hyperglycemia, hyperinsulinemia, hyperlipidemia, hyperleptinemia, and impaired endothelium-dependent vasodilation) compared to rats fed on standard chow. The hypothesis was that short-term HFD would induce early signs of renal disease. Young male Sprague-Dawley rats were fed either HFD (60% fat) or standard chow (5% fat) for six weeks. Morphology was determined by measuring changes in renal mass and microstructure. Kidney function was measured by analyzing urinary protein, creatinine, and hydrogen peroxide (H_2_O_2_) concentrations, as well as plasma cystatin C concentrations. Renal damage was measured through assessment of urinary oxDNA/RNA concentrations as well as renal lipid peroxidation, tumor necrosis factor alpha (TNF*α*), and interleukin 6 (IL-6). Despite HFD significantly increasing adiposity and renal mass, there was no evidence of early stage kidney disease as measured by changes in urinary and plasma biomarkers as well as histology. These findings suggest that moderate hyperglycemia and inflammation produced by short-term HFD are not sufficient to damage kidneys or that the ketogenic HFD may have protective effects within the kidneys.

## 1. Introduction

Diabetes is currently the leading cause of 44% of newly diagnosed cases of renal failure [1] as sustained hyperglycemia damages the filtration membranes of the kidneys resulting in albuminuria and proteinuria. In fact, proteinuria is often used as a surrogate marker for renal disease [2–4]. As the incidence of diabetes continues to increase throughout the world, rates of albuminuria and diabetic kidney disease (DKD) are likewise expected to increase [2].

The mechanisms leading to the development of chronic kidney disease (CKD) in subjects with metabolic syndrome, however, are not as well-understood [5]. According to the World Health Organization, the criteria for diagnosing metabolic syndrome indicate that an individual must have insulin resistance, type 2 diabetes, impaired glucose tolerance, or impaired fasting glucose along with two additional criteria: abdominal or overall obesity, dyslipidemia, hypertension, or microalbuminuria [6]. These characteristics of metabolic syndrome also increase the risk of developing cardiovascular disease and diabetes, which subsequently increase the risk of renal dysfunction and failure [6]. Foster et al. [7] examined data from the Framingham Heart Study that explored the relationship between renal fat accumulation, hypertension, and CKD. Participants were assessed for renal fat by computed tomography. Positive associations were identified between fatty kidney and hypertension and between fatty kidney and CKD. After adjusting for visceral adipose tissue, no association was found between fat infiltration in the kidney and diabetes. These data indicate that there may be an independent association between hypertension, renal fat accumulation, and CKD [7].

Deji et al. [5] showed that feeding 6-week-old mice a high fat diet (60% fat) for 12 weeks increased body mass, plasma glucose, insulin, and triglyceride concentrations and induced hypertension and kidney disease as evidenced by increased albuminuria, alterations in renal morphology, and renal lipid accumulation. Similarly, Altunkaynak et al. [8] demonstrated that feeding adult female Sprague-Dawley rats a moderate fat diet (30% fat) for 12 weeks caused the animals to become overweight and to develop increased kidney masses and volumes with significant morphological changes indicative of renal disease. Moreover, renal fat accumulation is evident with even moderate fat intake in rabbits (10% corn oil + 5% lard for 8–12 weeks) [9]. Stemmer et al. [10] compared lean chow-fed Wistar rats with HFD-fed rats that were either sensitive or partially resistant to diet-induced obesity after eleven months of HFD (40% butter fat). A positive correlation was established between levels of adiposity and the severity of renal damage. Their results indicate that lipotoxicity is not a strong contributor to renal dysfunction as they found that plasma triglyceride and free fatty acid as well as renal triglyceride levels did not differ significantly between the treatment and control groups [10].

While long-term feeding protocols are found in the literature, the effects of shorter-term feeding protocols to mimic early onset pathological changes within the kidney following high fat feeding are limited. A variety of rodent models have been used to investigate the effects of consuming HFD over the course of eight weeks to 11 months [5, 8, 10, 11], and although the effects of HFD on kidney morphology have been studied at four weeks [12], to our knowledge there are currently no studies that have examined the effects of shorter-term intake of high fat (as opposed to combined high carbohydrate, high fat) diets on renal function. For this reason, the present study was designed to examine the short-term effects of feeding rats a HFD (60% fat) for six weeks on renal morphology and biomarkers of function. Prior studies have shown that young (1.5-month-old) male Sprague-Dawley rats fed on a HFD (60% kcal from fat) for as few as six weeks develop indices of metabolic syndrome and cardiovascular disease: increased visceral adiposity, hyperglycemia, impaired glucose tolerance, hyperleptinemia, systemic inflammation (plasma TNF*α*), lipid peroxidation (TBARS), and impaired endothelium-dependent vasodilation [13, 14]. Other studies have shown that increased visceral adiposity, endothelial dysfunction, hypertension, inflammation, and oxidative stress (characteristics shared by these animals) increase blood pressure in the kidneys and promote renal damage, although this has yet to be examined in the 6-week HFD rat model. Therefore, exploring the effect of short-term HFD on the kidneys will fill a gap in the literature and allow for a better understanding of early pathological changes that occur due to consuming a HFD. It was hypothesized that six weeks of high fat intake (60% fat) would lead to early stages of renal disease in 1.5-month-old male Sprague-Dawley rats, as evidenced by morphological and functional changes in the kidney, compared to control rats on a standard rodent chow diet (5% fat).

## 2. Materials and Methods

### 2.1. Animal Models

Prior studies in our laboratory have shown that feeding young male (1.5-month-old) Sprague-Dawley rats (140–160 g body weight, Harlan Teklad Industries (Madison, WI, USA)) a HFD (60% kcal from fat; Cat. number D12492, Research Diets Inc., New Brunswick, NJ, USA) for six weeks results in symptoms associated with metabolic syndrome including increased adiposity, hyperleptinemia, hyperglycemia, endothelial dysfunction, and hypertension in comparison to animals fed on standard rodent chow [13, 14]. The current study examined isolated kidneys, plasma, and urine samples from a subset of animals examined in these prior IACUC-approved studies from September 2009 to May 2010. Blood samples were obtained via cardiac puncture and centrifuged at 13,000 rpm at 4°C for 15 minutes to separate formed elements from the plasma, which was then stored at −80°C until analyses. Urine was collected with a 25-gauge needle inserted directly into the bladder. The urine was then centrifuged at 13,000 rpm at 4°C for 10 minutes to remove debris and the supernatant was then stored at −80°C until analyses. Following a midline laparotomy, kidneys were removed and stored at −80°C until analyses while a separate subset was embedded in Optimal Cutting Temperature (OCT; Cat. number 4583; Sakura Finetek USA, Inc., Torrance, CA, USA) compound prior to freezing in isopentane cooled by liquid nitrogen and storing at −80°C.

### 2.2. Morphometrics

Animal morphometrics (body mass, epididymal fat pad mass, waist circumference, and tail length) and renal tissue masses were measured. Tail length is used as marker of overall growth in rats [15]. Epididymal fat pad mass was used to determine variations in adiposity between HFD and chow-fed rats because the epididymal fat pad can be easily removed from the animal objectively. Moreover, recent studies have shown that this fat pad is highly correlated (*R* ^2^ = 0.94) with total fat volume from the base of the skull to the distal tibia as measured by* in vivo* microCT scans [16].

A subset of frozen kidneys (*n* = 4 chow and *n* = 5 HFD rats) embedded in OCT compound were sectioned using a cryostat (Leica Biosystems CM1950; Buffalo Grove, IL, USA) and sections (14 *μ*m) were collected onto (+)-glass microscope slides (Histobond© adhesive slides, VWR VistaVision, Radnor, PA, USA). Sections were allowed to air-dry for 10 mins and were then fixed with 10% formalin for an additional 10 mins. Following 3-4 washes with tap water, sections were stained with Mayer's hematoxylin (Cat. number 6194A16; Hardy Diagnostics, Santa Maria, CA, USA) for 2 mins and then rinsed with warm tap water. The slides were then dipped in acid alcohol (0.5% HCl in 70% ethanol) and washed in Scotts Tap Water Substitute (Cat. number 26070-06; Electron Microscopy Sciences, Hatfield, PA, USA) for 5 mins followed by a rinse with deionized water. The sections were then counterstained with Eosin Y (Cat. number IS4054; Aldon Corporation, Avon, NY, USA) for 3 mins followed by a rinse with tap water and coverslips were mounted onto the glass microscope slides using SHUR/Mount (Cat. number LC-W; Triangle Biomedical Sciences, Durham, NC, USA). Sections were viewed using a light microscope (Olympus BX50) and images collected with an Olympus DP70 camera (Melville, NY, USA) to assess morphology.

### 2.3. Biomarkers of Renal Function

Measuring the excretion of total proteins in the urine, proteinuria, is a marker of early renal disease [3, 4]. In fact, multiple studies have found a stronger association between proteinuria and renal disease outcomes than any other tested factors [4]. Moreover, clinical studies on humans have identified proteinuria as among the first clinical symptoms of kidney damage [17, 18]. Total urinary protein concentrations were measured on a random set (*n* = 8/group) using the Bradford technique (Cat. number 500-0006; Bio-Rad, Hercules, CA, USA). Urinary creatinine concentrations were measured on the same set of urine samples (*n* = 8/group) using an available kit (Cat. number CR01; Oxford Biomedical Research, Rochester Hills, MI) and values were used to calculate the protein : creatinine ratio. Interestingly, creatinine clearance is also correlated with risk of renal failure [19].

Cystatin C produced by nucleated cells is filtered by the glomerulus and then catabolized by tubular cells in the kidney [20]. For this reason, plasma concentrations of cystatin C can be used as a biomarker of glomerular filtration rate [20]. Urinary changes in cystatin C can be used as markers of acute renal injury, although not often used in CKD assessment [21]. Serum creatinine concentrations are likewise often assessed as a marker of renal function; however, more recent studies indicate that serum or plasma cystatin C is a stronger, and more consistent, surrogate indicator of glomerular filtration rate and renal function [19–27]. Concentrations of cystatin C in the serum increase as much as 1-2 days prior to serum creatinine in conditions of acute kidney injuries [21] as well as in individuals with type 2 diabetes who have normoalbuminuria [28]. While serum creatinine levels can be affected by sex, age, diet, muscle mass, and body mass, cystatin C levels are independent of gender, muscle mass, and malignancy [21, 27]. Plasma cystatin C was therefore measured on a subset of samples (*n* = 10 per group) using an available ELISA kit (Cat. number MSCTCO; R&D Systems, Minneapolis, MN, USA) according to the manufacturer's protocol.

Urinary H_2_O_2_, a biomarker of inflammation, oxidative stress, and renal function, was likewise measured on a subset of samples (*n* = 6 per group) using an available kit (Cat. number ab102500; Abcam, Cambridge, MA, USA). Creatinine concentrations were measured from the same urine samples (Cat. number CR01; Oxford Biomedical Research, Rochester Hills, MI, USA) and the urinary H_2_O_2_ : creatinine ratio was calculated. Urinary and tissue H_2_O_2_ levels are often examined in studies of renal function. The increased renal perfusion pressure that is associated with hypertension has been found to elevate excretion of H_2_O_2_ [29]. Since H_2_O_2_ can stimulate proteinuria, it is sometimes used as an indicator of the initiation of renal pathology [30].

### 2.4. Inflammatory Markers

Chronic inflammation is often associated with multiple disease states and has been hypothesized to contribute to both morbidity and mortality in CKD patients [31]. Studies on animals fed on HFD have found lipid accumulation in glomeruli and proximal tubules within the kidneys along with increased expression of inflammatory markers, particularly tumor necrosis factor alpha (TNF*α*) and interleukin-6 (IL-6) [5, 32]. TNF*α* production is elevated with high fat intake and may in part cause insulin resistance [33] and both inflammatory cytokines can predict risk for chronic kidney disease [32]. Briefly, a subset of kidneys from both chow and HFD rats were transferred to a ground glass homogenizer containing Tris-HCI buffer (10 mM Tris (pH 7.6; Cat. number 161-0716; BioRad, Hercules, CA, USA), 1 mM EDTA, 1% triton X-100, 0.1% sodium deoxycholate, 0.03% protease inhibitor cocktail (Cat. number P2714; Sigma-Aldrich, St. Louis, MO, USA), and 1 mM phenylmethanesulfonyl fluoride (PMSF)). Samples were then centrifuged at 14,000 rpm for 10 minutes at 4°C to remove insoluble debris. Total protein concentrations of the supernatants were determined using the Bradford Technique (Cat. number 500-0006; Bio-Rad). Each sample (100 *μ*g total protein) was mixed with 6 *μ*L 5x sample buffer (0.6 mL 1 M Tris-HCl, pH 6.8; 5 mL 50% glycerol; 2 mL 10% sodium dodecyl sulfate; 1 mL 1% bromophenol blue; 0.9 mL deionized water and 2% *β*-mercaptoethanol as a reducing agent) and then boiled for 3 minutes. The mixture was then resolved using 4–15% gradient Tris-HCl SDS-PAGE gels (Cat. number 456-1083; Bio-Rad) and transferred to PVDF membranes (Cat. number 152-0176; Bio-Rad) for 90 minutes at 200 V. Membranes were then blocked for two hours in Tris buffered saline containing 0.05% Tween 20, 3% BSA fraction V, and 5% nonfat milk followed by an overnight incubation at 4°C in primary antibodies for IL-6 (1 : 250; Cat. number ab6672; Abcam, Cambridge, MA, USA), TNF*α* (1 : 500; Cat. number 11948; Cell Signaling, Danvers, MA, USA), or the loading control beta-actin (1 : 2000; Cat. number ab8227; Abcam, Cambridge, MA, USA). Primary antibodies were prepared in Tris buffered saline with 0.05% Tween 20 (TTBS). Membranes were then exposed to anti-rabbit IgG secondary antibody (1 : 1000 for IL-6 and TNF*α* and 1 : 2000 for beta actin; Cat number 7074S; Cell Signaling Technology, Danvers, MA) in TTBS for one hour at room temperature followed by exposure to Pierce enhanced chemiluminescence western blotting substrate (Cat. number 32279; Thermo Scientific, Rockford, IL, USA) for 1 min. Immunoreactive bands were visualized by exposure to X-ray film (Cat. number 34090; Thermo Scientific, Rockford, IL, USA) and analyzed using NIH ImageJ software. Protein expression of IL-6 and TNF*α* were normalized to beta-actin to determine the level of IL-6 (*n* = 5 chow and 6 HFD animals) or TNF*α* (*n* = 8/group) in each kidney sample.

### 2.5. Oxidative Stress

Kidneys are especially susceptible to oxidative stress due to a high concentration of long-chain polyunsaturated fatty acids, which easily undergo lipid peroxidation when exposed to reactive oxygen species [34]. Lipid peroxidation forms malondialdehyde as an end product, which can be detected via a thiobarbituric acid reactive substance (TBARS) assay, a marker of oxidative damage that is often examined. A subset of samples (*n* = 5/group) were used to evaluate renal lipid peroxidation using a commercially available TBARS assay kit (Cat. number 0801192; ZeptoMetrix Corporation, Buffalo, NY, USA).

8-Hydroxy-2′-deoxyguanosine (8-OHdG) is a sensitive biomarker of oxidative stress in tissue and bodily fluids and is a major product of damage caused by oxidative stress to DNA. 8-OHdG is produced by enzymatic cleavage of the guanine base, the base most prone to oxidation. Plasma samples (*n* = 10 chow; *n* = 11 HFD) were filtered with a 30 kDa ultrafilter (Cat. number UFC503096; EMD Millipore, Billerica, MA, USA) and 8-hydroxyguanosine, 8-OHdG, and 8-hydroxyguanine were examined as biomarkers of oxidative DNA and RNA damage using a commercially available kit (Cat. number 589320; Cayman Chemical, Ann Arbor, MI, USA), as oxidative stress has been shown to be elevated during early renal failure.

### 2.6. Statistical Analyses

Data are expressed as mean ± SEM. Statistical analyses were computed using SigmaPlot (Systat Software Inc., Version 13.0; San Jose, CA, USA). Data were tested for normality and then analyzed using Student's *t*-tests or the Mann-Whitney *U* tests, as appropriate. *p* values of ≤ 0.05 were considered significant.

## 3. Results

### 3.1. Morphometrics

Although the HFD rats tended to weigh more than the chow-fed rats, this difference was not statistically significant for the animals examined in this study (Table 1). Rats fed on a HFD demonstrated significantly increased epididymal fat pad mass compared to chow-fed animals, establishing that adiposity was increased by the HFD (Table 1). The waist circumferences of the HFD animals were significantly increased when compared to chow-fed controls, indicating that the HFD increased abdominal adiposity (Table 1). Tail lengths, a marker of overall growth, were not significantly different (Table 1). The renal masses of the HFD rats were significantly greater compared to the renal masses of animals in the chow group (Table 1).

**Figure 1:**
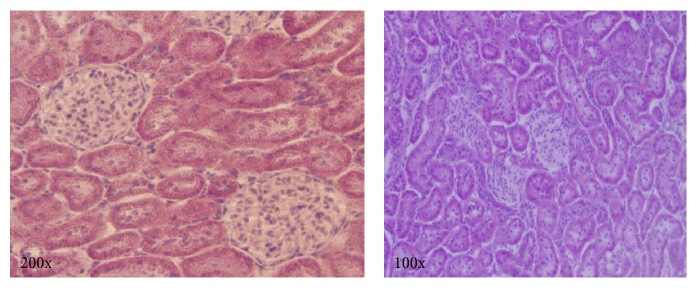
Representative images of hematoxylin and eosin stained renal tissue sections from HFD animals. Hematoxylin and eosin staining shows no signs of morphological damage.

Morphological analyses using hematoxylin and eosin-stained tissue sections showed no structural differences between the chow and HFD groups, indicating that although the mass of the HFD kidneys was increased, damage to the microstructure of the kidneys was not evident (Figure 1).

### 3.2. Biomarkers of Renal Function

The urine protein : creatinine ratios of the chow and HFD rats did not differ significantly, indicating that the filtering ability of the HFD rat kidneys was not damaged (Table 2). There was also no difference in the urine creatinine concentrations of the two groups (Table 2). Although HFD plasma cystatin C concentrations tended to be higher than chow rats, the difference was not sufficient to merit statistical significance (Table 2). Urinary hydrogen peroxide concentrations were significantly increased in the HFD animals. However, the H_2_O_2_ : creatinine ratios were not significantly different between the chow and HFD groups (Table 2).

### 3.3. Inflammatory Markers

**Figure 2:**
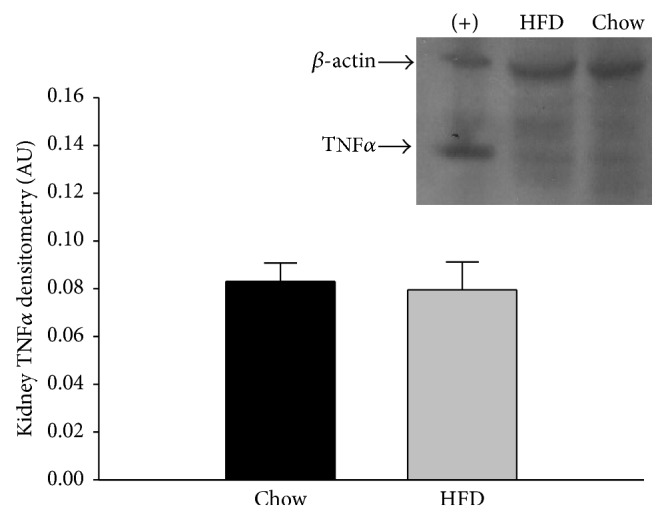
Renal tissue TNF*α* protein expression in chow and HFD rats. There was no difference in the TNF*α* expression of the two diet groups (*n* = 8/group). Retroperitoneal adipose tissue from a HFD rat was used as a positive control (+) and is shown in the first column. Data were analyzed by Student's *t*-tests and are expressed as means ± SEM. *p* = 0.803.

**Figure 3:**
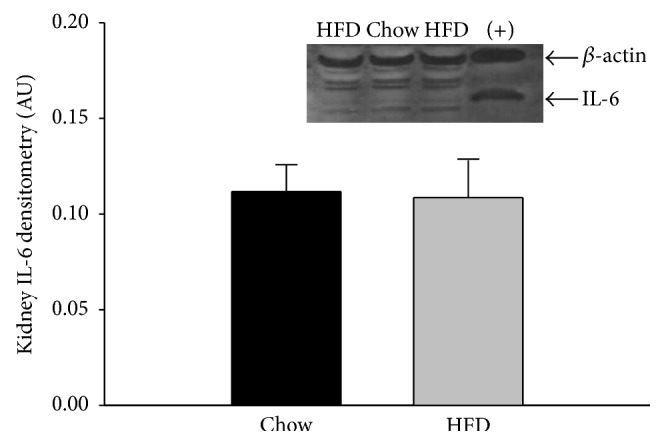
Renal tissue IL-6 protein expression in chow and HFD rats. There was no difference in the IL-6 expression of the two diet groups (*n* = 5 chow and 6 HFD rats). Retroperitoneal adipose tissue from a HFD rat was used as a positive control (+) and is shown in the last column. Data were analyzed by Student's *t*-tests and are expressed as means ± SEM. *p* = 0.903.

Western blot analyses of renal tissue established no significant difference in the TNF*α* protein expression of the chow and HFD rat kidneys (Figure 2). Quantification of western blots of IL-6 expression in renal tissues likewise showed no difference between the kidneys from chow and HFD rats (Figure 3).

### 3.4. Oxidative Stress Markers

Renal TBARS, a measure of tissue oxidative stress via lipid peroxidation, was not significantly elevated in the HFD rats (Table 2). Plasma levels of oxidized DNA and RNA, quantified as plasma levels of multiple oxidative stress markers including 8-hydroxyguanosine from RNA, 8-OHdG from DNA, and 8-hydroxyguanine, were examined. There were no significant differences between the chow and HFD rat groups (Table 2).

## 4. Discussion

Young (1.5-month-old) male Sprague-Dawley rats fed on HFD (60% fat) for six weeks developed significant increases in waist circumference and epididymal fat pad mass along with a trend towards increased body mass (Table 1). There was no significant difference in the tail lengths of the animals on the HFD and chow diets (Table 1), indicating that the animals were of similar body size. These data also indicate that the rats used for the present study developed increased adiposity. The use of an animal model that mimics the effects of metabolic syndrome and prediabetes is consistent with prior studies of HFD intake by both mice and rats [5, 8, 33, 35–39]. Previous studies from our laboratory also demonstrate that 6 weeks of HFD results in hyperglycemia, impaired glucose tolerance, hypertension, endothelial dysfunction, oxidative stress (plasma TBARS), and inflammation (plasma TNF*α*) in these animals, indicating that the HFD rats are also models of metabolic syndrome [13, 14].

The renal masses of the HFD rats examined in the present study were significantly higher than the chow rats (Table 1). The lack of difference in tail lengths (Table 1) signifies that the increased mass was not caused by overall growth of the animals and instead suggests structural damage to the kidneys. Altunkaynak et al. [8] found similar increases in body and renal masses of rats fed on HFD (30% fat) for 12 weeks. The increased renal mass may be attributed to hyperglycemia as proliferation and hypertrophy of mesangial cells and thickening of the glomerular basement membrane promote increased renal mass [40] along with vasodilation, inflammation, and increased connective tissue [8]. Despite the increased overall mass of the kidneys, hematoxylin and eosin staining of the HFD kidneys showed no evidence of morphological damage (Figure 1). This is in contrast to long-term studies of HFD that have been shown to cause morphological renal changes such as glomerular capillary dilation, enlarged lumens in the tubules and Bowman's capsule, amassing of extracellular proteins, nephron degradation, glomerular membrane thickening, glomerulosclerosis, renal and tubular interstitial cell necrosis, and shortened tubular epitheliums [5, 8, 41].

Proteinuria indicates that the filtering ability of the glomerulus is compromised and allowing proteins, which normally remain in the blood, to be passed through the filtration membrane and excreted. In evaluating renal function, albumin is sometimes specifically examined; however, by studying total protein excretion, a wider picture of filtration ability can be ascertained. Proteinuria, specifically, can be used as a marker of endothelial dysfunction as well as a surrogate outcome for renal disease progression. Urinary protein concentrations were normalized to urinary creatinine concentrations in order to account for variations in urine output [42]. Creatinine is also filtered by the kidneys and was therefore evaluated for differences between the HFD and chow-fed groups. Ruggiero et al. [36] found no significant changes in creatinine levels after C57BL mice consumed a 45% fat diet for sixteen weeks; the authors mentioned this indicated early stages of renal damage without severe functional damage (Table 2). In the present study, no statistically significant differences were observed between the urinary protein to creatinine ratio for animals ingesting the HFD as compared to the chow-fed animals (Table 2), indicating that the filtration abilities of the glomeruli were not damaged by the HFD or at least not to the extent of allowing large proteins to be excreted. With a longer duration feeding protocol, research suggests that the HFD may result in sustained elevations in blood glucose concentrations further leading to increased stress on the kidneys.

Plasma cystatin C is considered a marker of early renal dysfunction and the lack of difference between the cystatin C concentrations of the HFD and chow-fed rats indicates that the six-week feeding protocol may not have been long enough to initiate functional renal damage (Table 2). The HFD group did develop higher concentrations of plasma cystatin C; however, this difference was not statistically significant (Table 2). Although cystatin C levels in plasma have been shown to be an early sensitive marker of renal dysfunction, these results indicate that the kidneys of the HFD-fed rats were not damaged enough so as to prevent cystatin C from being filtered [22–24, 43]. Muntner et al. [44] demonstrated that serum cystatin C can be elevated in subjects who do not exhibit signs of micro- or macroalbuminuria or kidney disease. Rather, the authors concluded that serum cystatin C was correlated with cardiovascular disease in overweight subjects [44]. Therefore, plasma cystatin C alone may not be an accurate estimation of GFR.

H_2_O_2_ is an early marker of oxidative stress and inflammation. Despite the increased renal perfusion pressure that is associated with hypertension and elevated H_2_O_2_ excretion, there was no significant difference in urinary H_2_O_2_ : creatinine ratio between the two groups (Table 2). Elevated H_2_O_2_ in the HFD group would have indicated early renal damage, as H_2_O_2_ has been found to be elevated prior to GFR decline, proteinuria, and more robust inflammatory and fibrotic responses following high fat feedings [30, 37, 38]. Overall, these results demonstrate that although prior studies indicate that the HFD rats develop plasma and vascular oxidative stress and inflammation in addition to endothelial dysfunction [13, 14], these pathologies were not sufficient to impair renal function.

Western blot analyses established no significant difference between the TNF*α* concentrations of renal tissue isolated from HFD and chow-fed rats (Figure 2). Although increased TNF*α* expression in adipose tissue has been shown to promote to insulin resistance in overweight and obese rodent models [45, 46], there was no indication that the HFD increased renal tissue expression of this inflammatory cytokine (Figure 2). There was also no increase in IL-6 expression (Figure 3), a proinflammatory cytokine. Other experimental models of renal injury indicate that increases in IL-6 secretion are not necessarily an integral step in progressive renal failure [47]. Overall, the lack of significant findings in markers of early renal dysfunction, such as proteinuria and urinary H_2_O_2_, supports the lack of increases in cytokine expression. Chronic increased IL-6 levels are an indication of continued TNF*α* production and activation of inflammatory pathways. Despite the controversy regarding the differing effects of IL-6 as a cytokine and myokine, it continues to be studied as a marker of inflammation in HFD-induced obesity. Studies on both overweight and obese women have found that serum TNF*α* and IL-6 levels were positively correlated [48] while Ozay et al. [35] found that HFD-induced obesity in male Wistar albino rats (22% fat for 12 weeks) increased plasma TNF*α* without altering plasma IL-6. Conversely, Kaya et al. [49] found that IL-6 levels in obese human subjects were higher in both plasma and adipose tissue compared to TNF*α*. Stemmer et al. [10] examined the effect of HFD (40% of calories from butter fat for 11 months) on male Wistar rats and the creation of an inflammatory renal environment. Their results indicate an increase of IL-6 and TNF*α* in retroperitoneal fat and in the kidneys, while there was no increase in circulating IL-6.

Previous studies of the 6-week HFD rats showed that plasma TBARS, a measure of whole body oxidative stress, was increased in comparison to chow-fed controls [13]. Despite the increased plasma TBARS, renal TBARS were not significantly different between chow and HFD-fed rats (Table 2). These results indicate that although the HFD rats exhibited systemic oxidative stress, oxidative stress within the kidneys is not yet evident. Moreover, no significant difference between oxidative DNA/RNA damage between the experimental and control groups was observed (Table 2). Oxidative stress, examined via TBARS and DNA/RNA oxidation, is positively correlated [50]. The lack of significant TNF*α* and IL-6 expression in renal tissue examined in the present study is consistent with the observed lack of oxidative stress within the kidneys as inflammation and oxidative stress are typically correlated [51].

## 5. Conclusions

In conclusion, aside from morphological changes, the only major statistically significant findings in this study were an increase in visceral adiposity and renal mass following a short-term HFD. This study is novel in that it is the first to examine a HFD for only six weeks; prior to this study, the shortest feeding protocol that is evident in the literature is four weeks at which point only morphological changes were examined as opposed to measures of renal function. Previous research conducted on the six-week HFD rat model showed evidence of metabolic syndrome including significant vascular oxidative stress and inflammation, endothelial dysfunction, hypertension, hyperglycemia, hyperleptinemia, and impaired glucose tolerance [13, 14].

It is possible that the methods used in this study might not have been sensitive enough to detect subtle changes in renal function. In prior studies by our laboratory, 6 weeks of HFD significantly increased plasma concentrations of the ketone beta-hydroxybutyrate from 4.72 ± 0.31 in chow rats to 7.18 ± 0.77 mg/dL in the HFD animals [14]. Considering that ketogenic diets can reduce renal responses to glucose, it is also possible that the HFD exerts a protective effect in the kidney [51]. This was shown in a prior study of mouse models of type 2 diabetes with nephropathy in which 8 weeks of consuming a ketogenic diet fully normalized albumin : creatinine ratios and the expression of stress-related genes [51]. Therefore, it is apparent from the current study that prolonged hyperglycemia and inflammation are likely necessary to induce significant changes within the kidneys or that HFD may exert some protective effects through increased generation of ketones.

## Figures and Tables

**Table 1:** Morphometric measurements.

Measure	Chow (*n*)	HFD (*n*)	*p* value
Body mass (g)	349.8 ± 7.98 (9)	376.4 ± 10.3 (8)	0.056
Epididymal fat pad mass (g)	3.49 ± 0.18 (9)	5.60 ± 0.38 (8)	**<0.001**
Waist circumference (cm)	16.59 ± 0.30 (9)	17.96 ± 0.27 (8)	**0.004**
Tail length (cm)	20.1 ± 0.30 (9)	20.6 ± 0.32 (8)	0.276
Renal mass (g)	1.05 ± 0.04 (9)	1.19 ± 0.04 (8)	**0.019**

Data expressed as mean ± SEM and analyzed by Student's *t*-tests.

**Table 2:** Plasma and urinary markers of renal functionand damage.

Measure	Chow (*n*)	HFD (*n*)	*p* value
Urine creatinine (mg/L)	765.0 ± 110.6 (8)	900.3 ± 137.4 (8)	0.456
Urine protein : creatinine ratio (AU)	6.01 ± 2.86 (8)	3.89 ± 1.31 (8)	0.574
Urine H_2_O_2_ (pM/*µ*L)	0.023 ± 0.003 (6)	0.048 ± 0.005 (6)	**0.002**
Urine H_2_O_2_ : creatinine ratio (AU)	0.014 ± 0.002 (6)	0.026 ± 0.007 (6)	0.142
Plasma cystatin C (*μ*g/mL)	14.35 ± 0.99 (10)	16.2 ± 1.12 (10)	0.233
Renal TBARS (mM/L)	26.9 ± 2.02 (5)	28.08 ± 0.80 (5)	0.600
Plasma oxDNA/RNA (pg/mL)	887 ± 145 (10)	1003 ± 188 (11)	0.634

Data expressed as mean ± SEM and analyzed by Student's *t*-tests with the exception of urine protein : creatinine ratio, which was analyzed by the Mann-Whitney *U* test.
